# Ultrasound cycloplasty: dynamic efficacy and long-term safety in glaucoma patients from northwest China

**DOI:** 10.3389/fmed.2026.1819637

**Published:** 2026-10-15

**Authors:** Ping Ge, Rui Shi, Qing-Qing Mu, Rui-Xue Zhang, Xiang-Xiang Yang, Yuan He

**Affiliations:** 1 Department of Ophthalmology, The Second Affiliated Hospital of Xi'an Medical University, Xi'an, China; 2 Department of Ophthalmology, The 986th Hospital of the Air Force, Xi'an, China; 3 Department of Ophthalmology, Shaanxi Provincial People's Hospital, Xi'an, China

**Keywords:** dynamic efficacy, glaucoma, minimally invasive glaucoma surgery, Northwest China, UCP

## Abstract

**Purpose:**

To comprehensively evaluate the dynamic efficacy of UCP in treating different types of glaucoma patients in Northwest China, systematically analyze treatment outcome variations across follow-up time points, and assess long-term safety profiles

**Methods:**

In a 1-year follow-up study, 24 patients (30 eyes) with various types of glaucoma were enrolled and underwent UCP treatment (10 sectors or 8 sectors). Follow-up visits were conducted at different postoperative time points (1 day, 1 week, 1 month, 3 months, 6 months, and 1 year). Follow-up assessments consisted of IOP measurements, IOP reduction from baseline, surgical success rate, 24-hour IOP fluctuation, pain score improvement, and complications. Furthermore, the correlation between IOP at the final follow-up and baseline IOP was evaluated.

**Results:**

1. The overall IOP in all patients significantly decreased after UCP compared to preoperative levels (*P* < 0.01). 2. UCP demonstrated significant long-term IOP-lowering effects in patients with various types of glaucoma, with a 59.91% reduction at 1 year postoperatively. The efficacy was particularly notable in patients with primary angle-closure glaucoma, achieving a surgical success rate of 96.67% at 1 year. 3. Preoperative baseline IOP showed a positive correlation with IOP reduction at 1 year postoperatively (*r*_s_ = 0.641, *P* < 0.01), indicating that higher baseline IOP was associated with greater IOP reduction. 4. UCP significantly improved IOP fluctuation, with the mean 24-hour IOP fluctuation decreasing from 10.6 preoperatively to 5.3 postoperatively (*P* = 0.001). 5. UCP exhibited acceptable safety, with significant reductions in pain scores (*P* < 0.05) and no severe complications such as hypotony or macular edema observed during follow-up.

**Conclusion:**

1. In glaucoma patients in the Northwest population, UCP demonstrates significant long-term and dynamic efficacy—the higher the baseline IOP, the more pronounced the IOP-lowering effect, with outstanding performance in stabilizing IOP fluctuations. 2. UCP exhibits acceptable safety and controllable complications. Postoperative IOP stabilizes by 3 months, allowing for gradual withdrawal of medications, making it a reliable treatment option for glaucoma.

## 1 Introduction

Glaucoma is the leading global cause of irreversible vision loss and the primary irreversible blinding eye disease, typically associated with elevated intraocular pressure (IOP) (1). Epidemiological projections estimate that global glaucoma cases will exceed 111.8 million by 2040 (2). Although glaucoma affects all racial groups, studies indicate higher prevalence and severity in African and Asian populations (2).

The therapeutic goal of glaucoma is to preserve visual function, primarily achieved through IOP reduction and neuroprotection (3). Clinical management involves pharmacological, laser, or surgical interventions, with surgery considered when target IOP is unmet or progressive visual field deterioration persists despite treatment (3). Glaucoma surgeries fall into two categories: filtration procedures to enhance aqueous outflow and cyclodestructive procedures to reduce aqueous production (3).

Ultrasound cycloplasty (UCP) is an emerging minimally invasive procedure that utilizes high-intensity focused ultrasound (HIFU) to induce coagulative necrosis in ciliary epithelium, thereby decreasing aqueous humor production and lowering IOP (4). Compared to conventional surgeries, UCP offers advantages such as precise targeting, quantifiable energy delivery, operational simplicity, and repeatability. First implemented in Europe in 2011 (5). UCP was introduced to China in 2017. However, due to its relatively short clinical application history in China, robust studies evaluating its efficacy and safety in Chinese glaucoma patients remain scarce. Notably, existing research predominantly limits follow-up to ≤6 months and focuses on single-timepoint IOP measurements.

This single-center study assessed UCP outcomes in Northwest Chinese glaucoma patients, evaluating long-term dynamic IOP changes, 24-hour IOP stability, pain scores, medication reduction, and complications. Our findings validate UCP's efficacy and safety in this population, filling critical gaps in long-term domestic data and advancing precision treatment approaches for glaucoma.

## 2 Subjects and methods

### 2.1 Ethics

This study strictly followed the ethical guidelines of the Declaration of Helsinki. The research protocol was approved by the Clinical Trial Ethics Committee of the Second Affiliated Hospital of Xi'an Medical University (Approval No. XZY202363), ensuring full compliance with ethical and legal standards. Prior to participation, all subjects were clearly informed about the study objectives, procedures, and potential risks, and they provided voluntary written informed consent. Throughout the study, medical ethical principles were rigorously upheld to protect participants' rights and safety. Additionally, all collected data were handled with strict confidentiality measures, used exclusively for scientific research purposes, and managed following standardized data protection protocols to safeguard privacy.

### 2.2 Inclusion criteria

#### 2.2.1 Diagnostic criteria

POAG Diagnostic Criteria: The diagnosis is based on a comprehensive assessment of IOP, optic nerve damage, and visual field defects, with the core characteristic being an open anterior chamber angle. Based on IOP levels, POAG can be classified into high-tension and normal-tension glaucoma (NTG). High-tension POAG is characterized by pre-treatment peak IOP on diurnal curve exceeding 21 mmHg, accompanied by typical glaucomatous fundus changes, including morphological abnormalities of the optic disc and retinal nerve fiber layer (RNFL) damage. Optical coherence tomography (OCT) can further clarify optic nerve damage, visual field testing reveals glaucomatous visual field defects, and gonioscopy confirms an open angle, while other secondary causes of elevated IOP must be excluded. NTG is defined by pre-treatment IOP and peak IOP on diurnal curve consistently ≤21 mmHg, with characteristic optic nerve damage and visual field defects, an open angle, and requiring the exclusion of other optic neuropathies with similar presentations (e.g., ischemic optic neuropathy) and conditions associated with pseudo-low tension (e.g., spondylarthropathy). Ancillary examinations include gonioscopy, fundus examination with OCT, visual field testing, and 24-hour IOP monitoring for comprehensive evaluation (6, 7).

PACG Diagnostic Criteria: Diagnosis requires evidence of angle closure, elevated IOP, optic nerve damage, and characteristic clinical manifestations. Gonioscopy typically confirms partial or complete angle closure, accompanied by significantly elevated IOP (>21 mmHg, often >40 mmHg in acute attacks). Fundus examination and OCT reveal optic disc changes (e.g., increased cup-to-disc ratio) and RNFL defects, while visual field testing shows characteristic visual field loss. Acute angle-closure glaucoma presents with sudden onset of eye pain, headache, decreased vision, halos, nausea, and vomiting; mydriasis and corneal edema are typical signs. Chronic angle-closure glaucoma has an insidious onset and slow progression, with gradual angle closure and accompanying optic nerve damage. Auxiliary examinations such as ultrasound biomicroscopy (UBM) and anterior segment OCT assess the angle structure, and corneal thickness measurement helps account for its potential influence on IOP readings (6, 7).

SG Diagnostic Criteria: This category of glaucoma results from other ocular or systemic diseases, medication use, or ocular injury, leading to elevated IOP and/or optic nerve damage. Diagnosis necessitates identifying a clear underlying cause combined with clinical presentation and ancillary test results. Patients typically exhibit elevated IOP (>21 mmHg) and optic nerve damage (e.g., increased cup-to-disc ratio, optic disc rim thinning, or RNFL defects), with characteristic visual field defects confirmed by visual field testing. Gonioscopy may reveal angle abnormalities such as angle closure, synechiae, or neovascularization. Specific etiologies include ocular inflammation (e.g., uveitis), trauma, long-term corticosteroid use, vascular diseases (e.g., neovascular glaucoma), lens abnormalities (e.g., dislocation or phacolysis), and tumors or post-surgical complications, among others (6, 7).

#### 2.2.2 UCP indications

Based on current literature and guideline findings, UCP has broad applicability and can be indicated for various types of glaucoma, including:

Primary Glaucoma: such as POAG and PACG cases where IOP is inadequately controlled by medication (6).
Secondary Glaucoma: Including neovascular glaucoma, traumatic glaucoma, and pseudoexfoliative glaucoma (6, 8–11).
Refractory Glaucoma: Defined as glaucoma where effective IOP control cannot be achieved through conventional filtering surgery or topical hypotensive medication, ultimately leading to progressive vision loss and visual field deterioration even to the stage of absolute glaucoma (12–17).
Ocular Hypertension and Early-to-Moderate Stage Glaucoma: Compared to advanced glaucoma, UCP may demonstrate superior clinical applicability in these earlier stages (18).
Glaucoma Patients Without Previous Glaucoma Surgery History: Studies indicate that UCP also shows favorable IOP-lowering effects in these patients (8, 11).

#### 2.2.3 Inclusion criteria

Target Population: no restrictions on age or gender.
General Health: medically stable and able to tolerate the procedure.
Diagnosis: glaucoma confirmed in the study eye undergoing UCP treatment.
Indication: meets UCP criteria with inadequate IOP control despite maximal medical therapy.
Consent: willingness to provide signed informed consent for UCP treatment.

### 2.3 Exclusion criteria

Poor systemic status: medically unfit for surgery.
Ocular structural abnormalities: including large filtering blebs, significant conjunctival scarring, or staphyloma.
Active ocular inflammation: history of active uveitis or other inflammatory eye diseases within the past 3 months.
Prior cyclodestructive procedures: any history of ciliary body ablation surgeries within the last 3 months (including but not limited to filtering surgery, minimally invasive glaucoma surgery, and aqueous drainage device implantation).
Confounding Surgeries: previous intraocular surgeries that may interfere with efficacy assessment (e.g., cataract surgery, vitreoretinal surgery).
Device Limitation: anatomically unsuitable for UCP probe placement.

### 2.4 Surgical procedure

A comprehensive preoperative evaluation and preparation must be conducted prior to surgery. First, a systematic collection of the patient's medical history should be performed, with particular attention paid to systemic conditions such as cardiovascular and cerebrovascular diseases. Regarding ocular conditions, details should be documented, including the onset time of eye diseases, major symptoms, accompanying manifestations, and past treatment experiences—such as ocular trauma, medication use, and surgical history. For systemic examinations, routine tests include hematological assessments (complete blood count, coagulation function, liver and kidney function, electrolytes), infection screening (transfusion-related tests), cardiac evaluation (electrocardiogram), and chest imaging (chest x-ray). For patients at risk of immune-related diseases, additional specialized tests such as rheumatology panels may be necessary. If required, further imaging studies—such as MRI of the sacroiliac joint, brain CT, or MRI—should be performed. Ophthalmic examinations must include 24-hour dynamic IOP monitoring to comprehensively assess fluctuations. Precise measurements of corneal diameter (“white-to-white” distance) and axial length—obtained using ocular A/B ultrasound, anterior and posterior segment OCT, and IOL Master—are critical for selecting the appropriate probe size (10 mm/11 mm/12 mm/13 mm) and determining surgical parameters such as frequency, energy output, and the number of activated sensors. Additionally, fundus photography, OCT, and visual field testing should be performed to evaluate the extent of optic nerve damage (19).

UBM plays a crucial role in preoperative evaluation. It provides clear visualization of the ciliary body's position, morphology, and structure, facilitating precise parameter measurements. During UBM examination, the scan must pass through the maximum corneal diameter (“white-to-white”), with panoramic UBM images captured in both horizontal and vertical axes to fully display bilateral ciliary processes, as shown in Figure 1. If multiple UBM scans fail to reveal prominent ciliary processes, this suggests potentially suboptimal UCP treatment outcomes. For probe selection, comprehensive consideration of axial length and corneal diameter data is essential, with computer-assisted fitting utilized to determine the optimal probe size. Inaccurate energy focal point positioning (e.g., excessive posterior displacement or localization at the iris root) can significantly compromise surgical efficacy, as illustrated in Figure 2.

**Figure 1:**
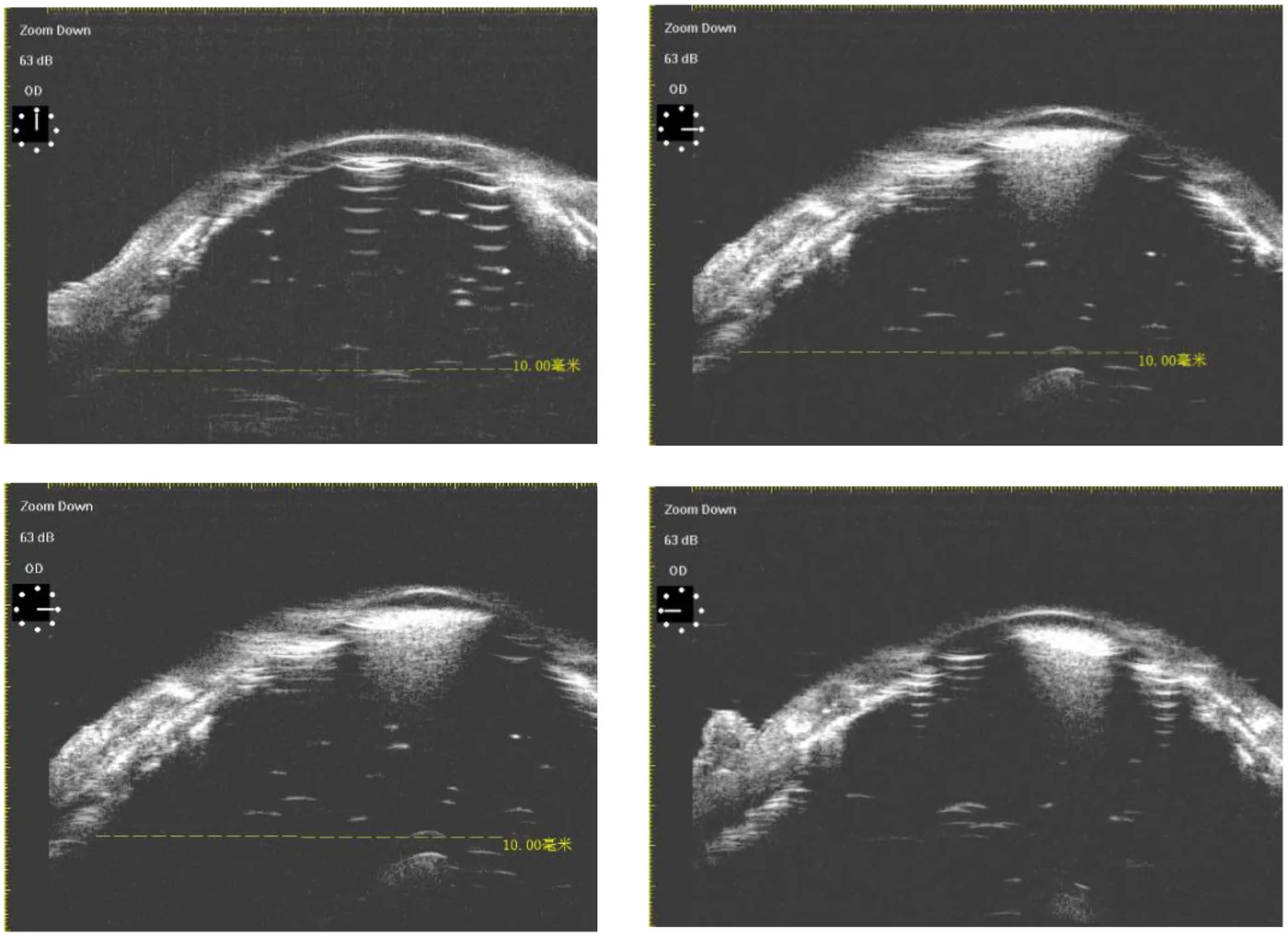
Preoperative UBM image of the patient.

**Figure 2:**
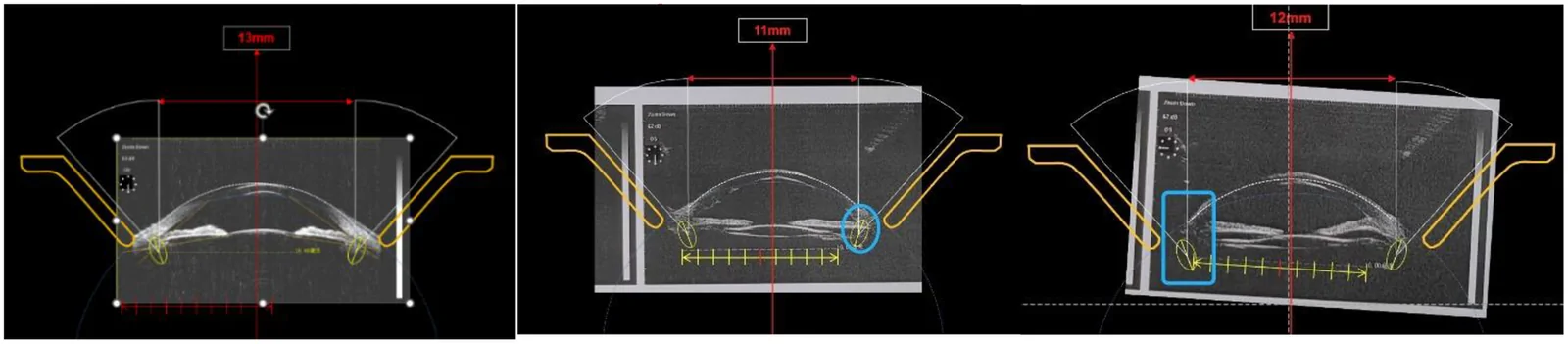
Fitting diagram of UCP probe.

The primary energy parameter for UCP is the acoustic energy delivered per treatment sector. The widely adopted and evidence-based protocol uses a fixed sonication time of 22 s with an acoustic power output of 2.3 Watts (W) per transducer, resulting in an acoustic energy of 50.65 Joules (J) per sector (20). This parameter primarily determines the thermal effect on the ciliary body and is the mainstay of most clinical studies (20). The selection of the sector number is empirically related to preoperative baseline IOP and the severity of glaucoma to balance efficacy with safety. 6 sectors: Baseline IOP <30 mmHg. 8 sectors: Baseline IOP ≥ 30 mmHg. 10 sectors: Baseline IOP ≥ 40 mmHg (often reserved for eyes with no light perception or very advanced disease requiring maximal IOP reduction). The nasal and temporal quadrants were deliberately left untreated in all procedures. This practice aims to avoid the ciliary nerves located in these areas, thereby significantly reducing postoperative pain. This is now standard in the protocol to improve patient comfort (10, 21). This study also adhered to this established protocol for energy and sector selection.

Prophylactic anti-inflammatory treatment should be initiated preoperatively, such as administering NSAIDs and corticosteroid eye drops in advance. Additionally, intraocular pressure-lowering measures should be implemented, including topical quadruple anti-glaucoma medication or, depending on the patient's condition, anterior chamber paracentesis. Nutritional therapy targeting corneal and optic nerve health should also be incorporated into the treatment plan.

The figures demonstrate (from left to right): Normal-sized probe, Undersized probe with energy focus at the iris root, Oversized probe with excessively posterior energy focus.

During the procedure, the patient should be placed in a supine position with the head tilted backward to ensure the ocular axis remains perpendicular to the ground. After administering peribulbar or topical anesthesia, carefully position the fixation ring in a centered manner, ensuring its circular edge is parallel to the corneal limbus while maintaining equal distance at all points. Conduct a vacuum pressure test using a clamping suction tube and release the clamp once testing is complete. Throughout the surgery, it is essential to maintain proper fixation ring alignment—ensuring stability, centration, and minimal tilt. Once the eye is securely fixed, activate the negative pressure system and insert the treatment probe into the ring. Perform the entire procedure in a fluid-filled environment using sterile balanced salt solution or saline. Continuously monitor and stabilize vacuum pressure throughout the operation to ensure safety. For treatment parameters, each sector should receive energy delivery for no more than 8 s, followed by a 20-second interval before proceeding to the next sector. In cases requiring 8 or 10 treatment sectors, rotate the probe after completing the first 6 sectors to treat the remaining zones effectively. Maintaining precise execution at each step ensures optimal surgical outcomes (22). The entire treatment process typically lasts 3 to 5 min.

Postoperatively, patients should routinely use nonsteroidal anti-inflammatory drugs (NSAIDs) or a combination of corticosteroid and antibiotic eye drops. Elevated IOP should be managed for 3 months: If IOP remains 18–21 mmHg, continue antiglaucoma medications. If IOP is 10–18 mmHg, consider tapering beta-blockers. If IOP falls below 10 mmHg, reduce medications cautiously and perform B-scan ultrasound to rule out choroidal detachment. Aptel, Gregorio, et al. have demonstrated that repeatable UCP treatment is effective and safe (23, 24).

All UCP procedures were performed by the same experienced senior ophthalmologist. The patient was placed in a supine position with slight chin elevation to align the visual axis perpendicular to the horizontal plane. Standard disinfection and sterile draping were performed. Anesthesia (retrobulbar, peribulbar, or general) was administered based on preoperative assessment. An eyelid speculum was inserted, followed by irrigation of the surgical eye with diluted povidone-iodine solution and thorough rinsing with normal saline. The UCP system was activated, and patient data were entered. The treatment sector was selected, followed by coupling the probe to the positioning ring. The positioning ring was centered to produce a uniform scleral whitening halo, ensuring proper probe alignment. The coupling cone was secured under vacuum, with an audible “click” indicating proper probe placement. Sterile balanced saline was injected into the gap between the ring and probe. Treatment was initiated via foot pedal (8 s per sector, with 20-second intervals). After treatment completion, the probe and positioning ring were removed. Anti-inflammatory ophthalmic ointment was applied to the conjunctival sac, and the eye was patched.

Postoperative patients should routinely use non-steroidal anti-inflammatory drugs or corticosteroid eye drops combined with antibiotic eye drops, while intraocular pressure management should be continued for up to three months—if the pressure remains between 18 and 21 mmHg, antiglaucoma medication should be continued; if it is between 10 and 18 mmHg, a gradual reduction of beta-blockers may be considered; and if it falls below 10 mmHg, medications should be tapered cautiously, and a B-scan ultrasound should be performed to rule out choroidal detachment.

### 2.5 UCP success criteria

The surgical success criteria in this study were based on the definitions by Aptel and Zhang (25, 26), specifically defined as follows:

**Primary Success Criteria:** IOP Reduction: Postoperative mean IOP reduction ≥20% from preoperative baseline IOP (arithmetic mean of 24-hour pre-op IOP measurements). Safety Threshold: IOP ≥5 mmHg at all follow-up visits. Complication-Free: No severe postoperative complications (e.g., sustained hypotony, choroidal detachment). Medication-Free: No requirement for IOP-lowering medications.
**Stratified Success Definitions:** Complete Success: IOP ≤21 mmHg without any IOP-lowering medications. Qualified Success: IOP ≤21 mmHg with fewer medications than preoperatively, OR IOP >21 mmHg but showing: ≥20% reduction from preoperative IOP AND absolute IOP ≥5 mmHg (without medications). Failure: Requirement for additional glaucoma surgery, OR failure to achieve IOP ≤21 mmHg despite using the same number of medications as preoperatively.

### 2.6 IOP measurement and mean amplitude of IOP excursion

IOP was measured using an I-care tonometer. Each measurement was performed three times, and the average value was calculated as the final IOP reading to minimize measurement error. IOP measurements included 24-hour dynamic monitoring. All measurements were conducted by the same ophthalmic technician.

The 24-hour intraocular pressure fluctuation was assessed using the Mean Amplitude of IOP Excursion (MAPE) (27). All measurements were performed by the same experienced ophthalmic technician to ensure consistency. Each patient underwent continuous 24-hour IOP monitoring, with three repeated measurements taken at each time point and averaged.

Calculation method for MAPE: **Drift Value (ΔIOP):** Compute the change in IOP between consecutive time points. **Standard Deviation (SD):** Calculate the SD of all IOP measurements across the 24-hour period. **Valid Excursions:** Identify ΔIOP values exceeding the SD as **significant fluctuations (valid excursions)**. **Final MAPE Value:** Compute the arithmetic mean of all valid excursions (see Figure 3). This methodology ensures standardized detection of clinically meaningful IOP fluctuations while minimizing measurement variability.

**Figure 3:**
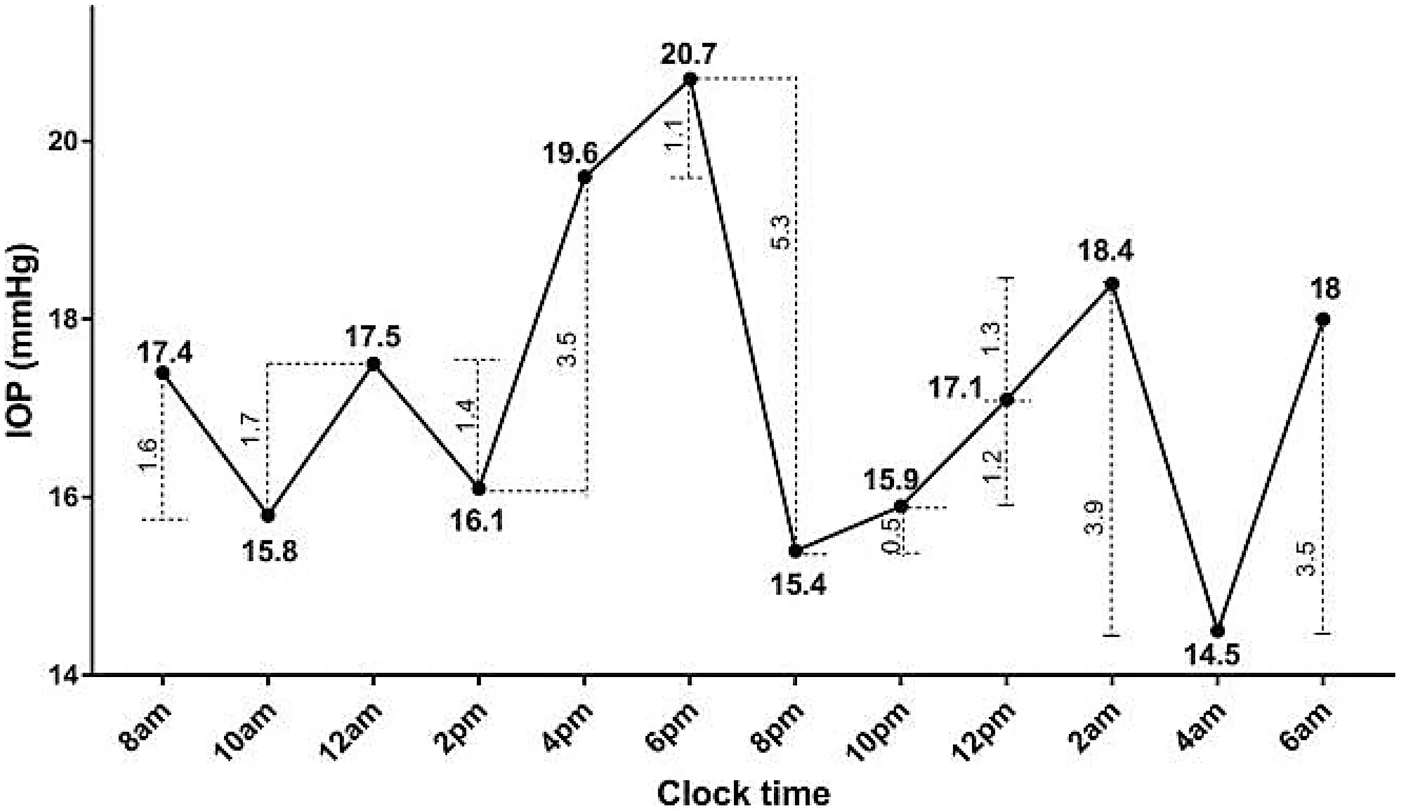
Calculation method of MAPE. Reprinted with permission from Zhai et al. (27). Copyright © 2021Springer Nature.

### 2.7 Statistical methods and sample size calculation

In the sample size estimation for this study, we utilized the statistical software PASS 2023. The calculation was based on the formula for comparing means between two independent samples, as follows:

$$n=\frac{2\times {\left(Z_{\alpha /2}+Z_{\beta }\right)}^{2}\times \sigma^{2}}{\Delta^{2}}$$

Where:

- *n* is the minimum required sample size per group.
- Z*α*/2 is the critical value from the standard normal distribution corresponding to the significance level (for a two-tailed test with *α* = 0.05, Z*α*/2 = 1.96).
- Z*β* is the critical value corresponding to the statistical power (for a power of 1 − *β* = 0.80, Z*β* = 0.842).
- *σ* is the overall standard deviation (based on literature reports, *σ* = 12%).
- The denominator is the preset effect size, representing the expected mean reduction in intraocular pressure (according to literature reports, the mean IOP reduction = 40%).

Substituting these parameters into the formula:

$$n=\frac{2\times {\left(1.96+0.842\right)}^{2}\times 12^{2}}{40^{2}}\approx 26$$

Therefore, the calculated minimum required sample size per group is 26 eyes. To account for potential loss to follow-up, data attrition, and follow-up errors, a final total of 30 eyes were included for statistical analysis. The sample size calculation confirms that the inclusion of 30 eyes provides sufficient statistical power to effectively detect efficacy differences attributed to UCP, ensuring the reliability and positive significance of the study's results.

Data analysis was performed using SPSS 26.0. Categorical variables were presented as frequencies (*n*) and percentages (%). Continuous variables were tested for normality using the Shapiro–Wilk test, with normally distributed data expressed as mean ± standard deviation (x¯ ± SD) and non-normally distributed data reported as median (Md) and interquartile range (IQR). For between-group comparisons of repeated measures, statistical methods were selected based on normality: Normally distributed data: Repeated-measures ANOVA (post-hoc LSD-t test). Non-normally distributed data: Friedman test (with Bonferroni-corrected Wilcoxon signed-rank tests). Correlation analysis: Pearson's test was applied for normally distributed data. Spearman's test was used for non-normally distributed data. The relationship between postoperative 1-year IOP reduction and preoperative baseline IOP was assessed via Spearman correlation analysis (*r* > 0 indicates positive correlation, *r* < 0 indicates negative correlation, *P* *<* *0.05 c*onsidered statistically significant). Comparisons of MAPE and pain scores before and after UCP were conducted using the Wilcoxon signed-rank test. Figures and charts were generated using GraphPad Prism 9.0. All analyses strictly adhered to the prespecified statistical protocol to ensure objective and reliable results.

### 2.8 Pain assessment

The intensity of ocular pain in patients was quantified using the Numerical Rating Scale (NRS), with detailed recordings of pain levels documented, as shown in Figure 4 (28). The scoring range is 0–10: 0 points represent no pain, recorded as Grade 0; 1–4 points represent mild pain, recorded as Grade I; 5–9 points represent moderate pain, recorded as Grade II; and 10 points represent severe pain, recorded as Grade III. This grading system provides a clear quantitative basis for evaluating pain levels.

**Figure 4:**
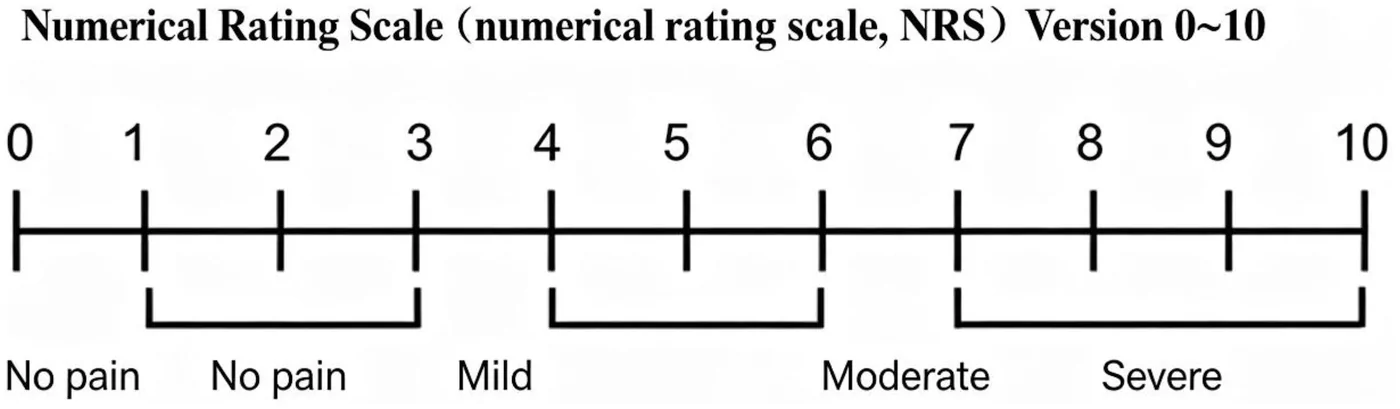
The numerical rating scale. Reprinted with permission from Wan et al. (28). Copyright © 2020 The Editorial Office of Chinese Journal of Pain Medicine.

## 3 Results

### 3.1 Demographic data

The study enrolled 24 glaucoma patients (30 eyes) from Northwest China, with a mean age of 51.5 ± 17.32 years. The cohort included 13 males (54.2%) and 11 females (45.8%), with 14 right eyes (46.7%) and 16 left eyes (53.3%) included in the analysis. The glaucoma subtypes were distributed as follows: Primary Angle-Closure Glaucoma (PACG): 10 eyes (33.3%), Primary Open-Angle Glaucoma (POAG): 8 eyes (26.7%), Neovascular Glaucoma (NVG): 4 eyes (13.3%), Secondary Glaucoma (SG): 8 eyes (26.7%), detailed demographic and baseline characteristics are summarized in Table 1.

**Table 1:** Patient demographic and clinical characteristics.

Characteristic category	Stratification/value	Number of eyes *n* (%)
Sex	Male	13 (54.2)
	Female	11 (45.8)
Laterality	Right eye	14 (46.7)
	Left eye	16 (53.3)
Diagnosis	PACG	10 (33.3)
	POAG	8 (26.7)
	NVG	4 (13.3)
	SG	8 (26.7)
Preoperative IOP (mmHg)	≤21	3 (10.0)
	22–29	4 (13.3)
	30–39	10 (33.3)
	≥40	13 (43.3)
BCVA (LogMAR)	No light perception	7 (23.3)
	Light perception	2 (6.7)
	Hand motion/counting fingers	3 (10.0)
	0.7–2.0	8 (26.7)
	＜0.7	10 (33.3)
Preoperative Medications (No.)	1.00	3 (10.0)
	2.00	1 (3.3)
	3.00	4 (13.3)
	4.00	22 (73.3)
Pain Level	No pain	11 (36.7)
	Mild	3 (10.0)
	Moderate	16 (53.3)

### 3.2 Efficacy

#### 3.2.1 IOP changes before and after UCP in all patients

To evaluate the overall IOP differences before and after UCP at various time points (1 day, 1 week, 1 month, 3 months, 6 months, and 1 year), Friedman test was performed due to the skewed distribution of IOP, and results were presented as median (interquartile range). The results showed that preoperative IOP was 40.5 (26, 48.5) mmHg in 30 eyes, while postoperative IOP at 1 day, 1 week, 1 month, 3 months, 6 months, and 1 year was 18 (12.8, 28.8), 14 (11.8, 19), 13 (11, 20.5), 15 (12, 19.3), 15 (12.8, 16), and 12 (11, 15) mmHg, respectively (*χ*² = 76.791, *P* < 0.0001). Bonferroni-corrected Wilcoxon signed-rank test revealed significantly lower IOP at all postoperative follow-up time points compared to preoperative baseline (*χ*² = 2.533, *χ*² = 3.800, *χ*² = 3.267, *χ*² = 3.200, *χ*² = 3.317, *χ*² = 4.300, all *P* < 0.0001) (Figures 5, 6). These results indicate statistically significant differences in IOP before and after surgery. Notably, the most substantial IOP reduction was observed at 1 year postoperatively (*χ*² = 4.300), with detailed data shown in Table 2.

**Figure 5:**
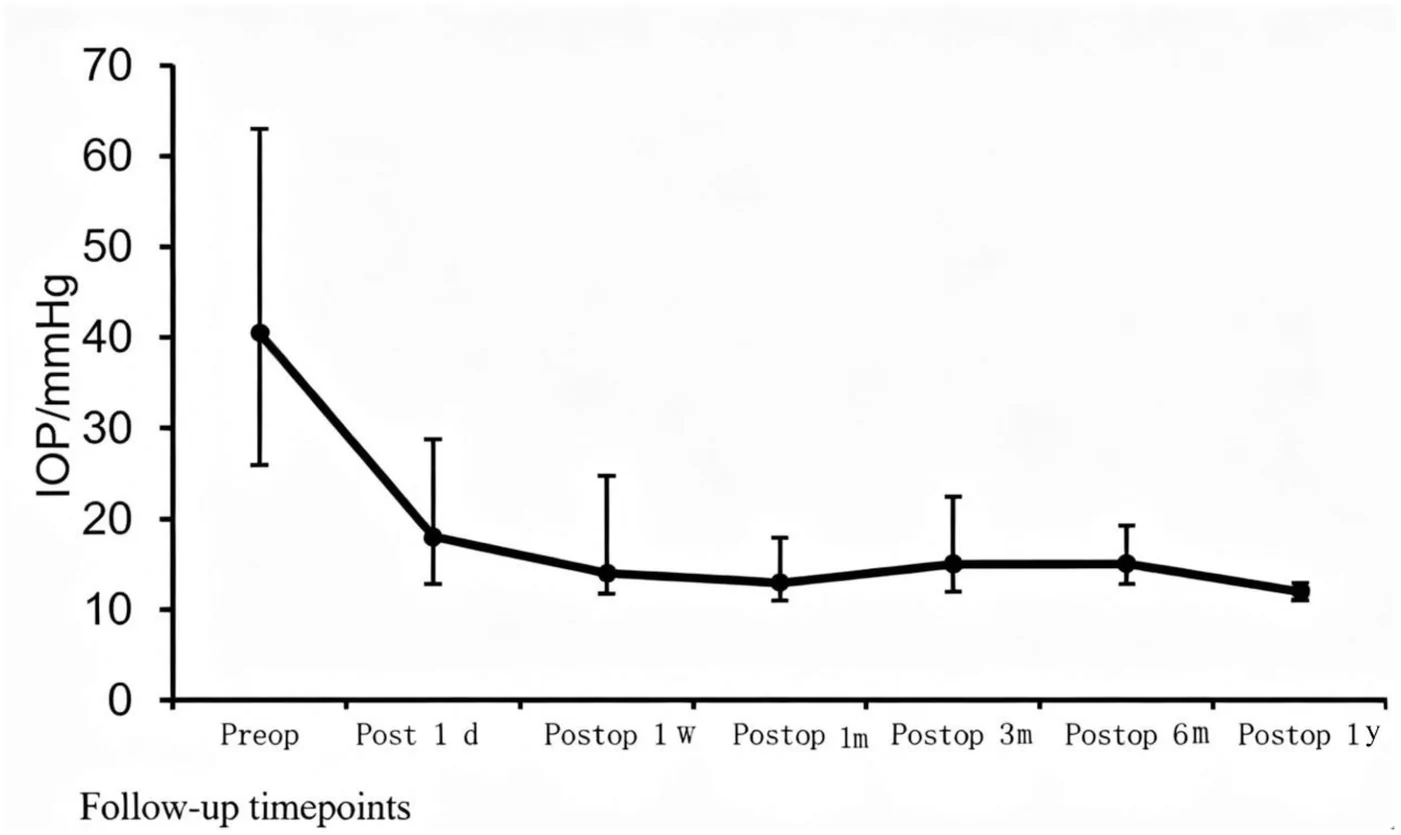
Changes in overall IOP before and after UCP.

**Figure 6:**
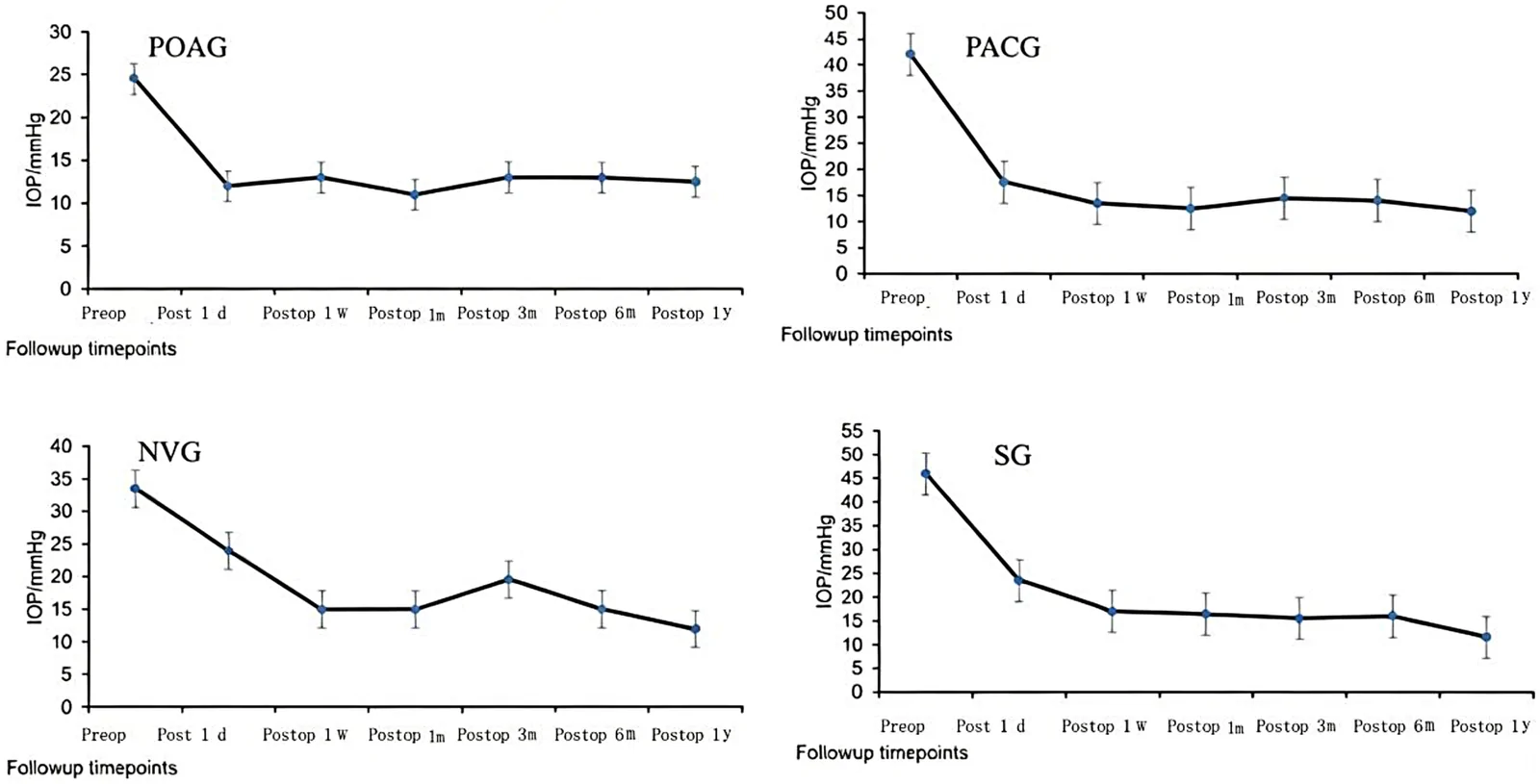
Pre- and post-UCP IOP changes across different glaucoma types.

**Table 2:** Results of nonparametric tests comparing IOP between postoperative time points and preoperative baseline.

Timepoint Comparison	*χ*² Value	*P* Value
Postop 1 day vs. Preop	2.533	<0.0001
Postop 1 week vs. Preop	3.800	<0.0001
Postop 1 month vs. Preop	3.267	<0.0001
Postop 3 months vs. Preop	3.200	<0.0001
Postop 6 months vs. Preop	3.317	<0.0001
Postop 1 year vs. Preop	4.300	<0.0001

Data followed non-normal distribution, thus nonparametric tests were used. Pairwise comparisons were Bonferroni-corrected. *P* < 0.05 indicates statistical significance.

#### 3.2.2 IOP changes before and after UCP in different types of glaucoma

This study included a total of 24 glaucoma patients (30 eyes), comprising 10 eyes with PACG, 8 eyes with POAG, 4 eyes with NVG, and 8 eyes with other types (SG).

Friedman test analysis of IOP changes in POAG patients (*n* = 8 eyes) demonstrated statistically significant differences (*χ*² = 20.094, *P* < 0.05) between preoperative median IOP [24.5 mmHg (15,37.8)] and postoperative values, which measured 12 mmHg [6,17.3] at 1 day, 13 mmHg [8,14.8] at 1 week, 11 mmHg [8,13.8] at 1 month (most significant reduction), 13 mmHg [10,16.5] at 3 months, 13 mmHg [12,15] at 6 months, and 12.5 mmHg [11,14] at 1 year, with Bonferroni-corrected Wilcoxon signed-rank tests showing significant reductions at 1 day (*χ*² = 3.625), 1 week (*χ*² = 3.688), 1 month (*χ*² = 4.250), and 1 year (*χ*² = 3.375) postoperatively (all *P* < 0.05).

Friedman test analysis comparing preoperative and postoperative IOP changes in PACG patients (*n* = 10 eyes) revealed statistically significant differences (*χ*² = 29.380, *P* < 0.05) between the preoperative median IOP [42 mmHg (36,47)] and postoperative values, measuring 17.5 mmHg [12.8,27.5] at 1 day, 13.5 mmHg [11.8,16.3] at 1 week (most significant reduction), 12.5 mmHg [11,20.8] at 1 month, 14.5 mmHg [11,16.3] at 3 months, 14 mmHg [13,16] at 6 months, and 12 mmHg [11,15.5] at 1 year, with Bonferroni-corrected Wilcoxon signed-rank tests demonstrating significant reductions at 1 week (*χ*² = 4.350), 1 month (*χ*² = 3.500), 3 months (*χ*² = 3.450), 6 months (*χ*² = 2.950), and 1 year (*χ*² = 4.300) postoperatively (all *P* < 0.05), confirming statistically significant IOP differences following UCP treatment in PACG patients.

For NVG patients, Friedman test analysis was performed to evaluate the significance of IOP changes between preoperative and postoperative time points (1 day, 1 week, 1 month, 3 months, 6 months, and 1 year). The results showed that the preoperative median IOP was 33.5 mmHg (27.5, 38) in 4 eyes. Postoperative IOP values were 24 mmHg (11, 33.3) at 1 day, 15 mmHg (10.3, 26.5) at 1 week, 15 mmHg (12, 30) at 1 month, 19.5 mmHg (13.8, 28.3) at 3 months, 15 mmHg (11.8, 19.1) at 6 months, and 12 mmHg (10.2, 13.5) at 1 year, with *χ*² = 14.172, *P* < 0.05. Bonferroni-corrected Wilcoxon signed-rank tests revealed that IOP was significantly lower than preoperative levels at 1 week (*χ*² = 3.375), 6 months (*χ*² = 4.125), and 1 year (*χ*² = 5.375) postoperatively (all *P* < 0.05). The results demonstrate significant IOP reduction following UCP treatment in NVG patients, with the most pronounced decrease observed at 1 year postoperatively (*χ*² = 5.375).

For SG patients, Friedman test analysis was conducted to evaluate the significance of IOP changes between preoperative and postoperative time points (1 day, 1 week, 1 month, 3 months, 6 months, and 1 year). The results showed that preoperative median IOP was 46 mmHg (42, 57.5) in 8 eyes, while postoperative IOP values were 23.5 mmHg (15, 32.5) at 1 day, 17 mmHg (12.3, 24) at 1 week, 16.5 mmHg (13, 37) at 1 month, 15.5 mmHg (12, 19.8) at 3 months, 16 mmHg (12.8, 16.8) at 6 months, and 11.5 mmHg (11, 15) at 1 year (*χ*² = 25.845, *P* < 0.05). Bonferroni-corrected Wilcoxon signed-rank tests revealed significantly lower IOP compared to preoperative levels at 1 week (*χ*² = 3.688), 6 months (*χ*² = 3.688), and 1 year (*χ*² = 4.813) postoperatively (all *P* < 0.05). The results indicate statistically significant IOP reductions following UCP treatment in SG patients, with the most pronounced decrease observed at 1 year postoperatively. Pre- and postoperative IOP changes in different types of glaucoma following UCP surgery are presented in Figure 6.

### 3.3 Magnitude of IOP reduction at different time points after UCP

Analysis of IOP reduction at various time points post-UCP demonstrated: Overall in 30 eyes, the IOP decreased by 44.41%, 55.42%, 53.02%, 52.99%, 55.81%, and 59.91% at 1d, 1w, 1m, 3m, 6m, and 1y respectively. By subtypes: POAG (8 eyes) showed reductions of 44.58%, 44.68%, 49.42%, 41.82%, 43.37%, and 46.01%; PACG (10 eyes) 50.37%, 65.99%, 62.61%, 63.27%, 62.82%, and 66.12%; NVG (4 eyes) 33.68%, 48.73%, 41.34%, 38.28%, 52.63%, and 63.25%; SG (8 eyes) 45.85%, 58.88%, 51.81%, 58.98%, 60.42%, and 64.74%. Results indicated maximum IOP reduction occurred at 1w post-op for POAG, while for overall cases and other glaucoma subtypes, peak reduction was observed at 1y post-op (Figure 7).

**Figure 7:**
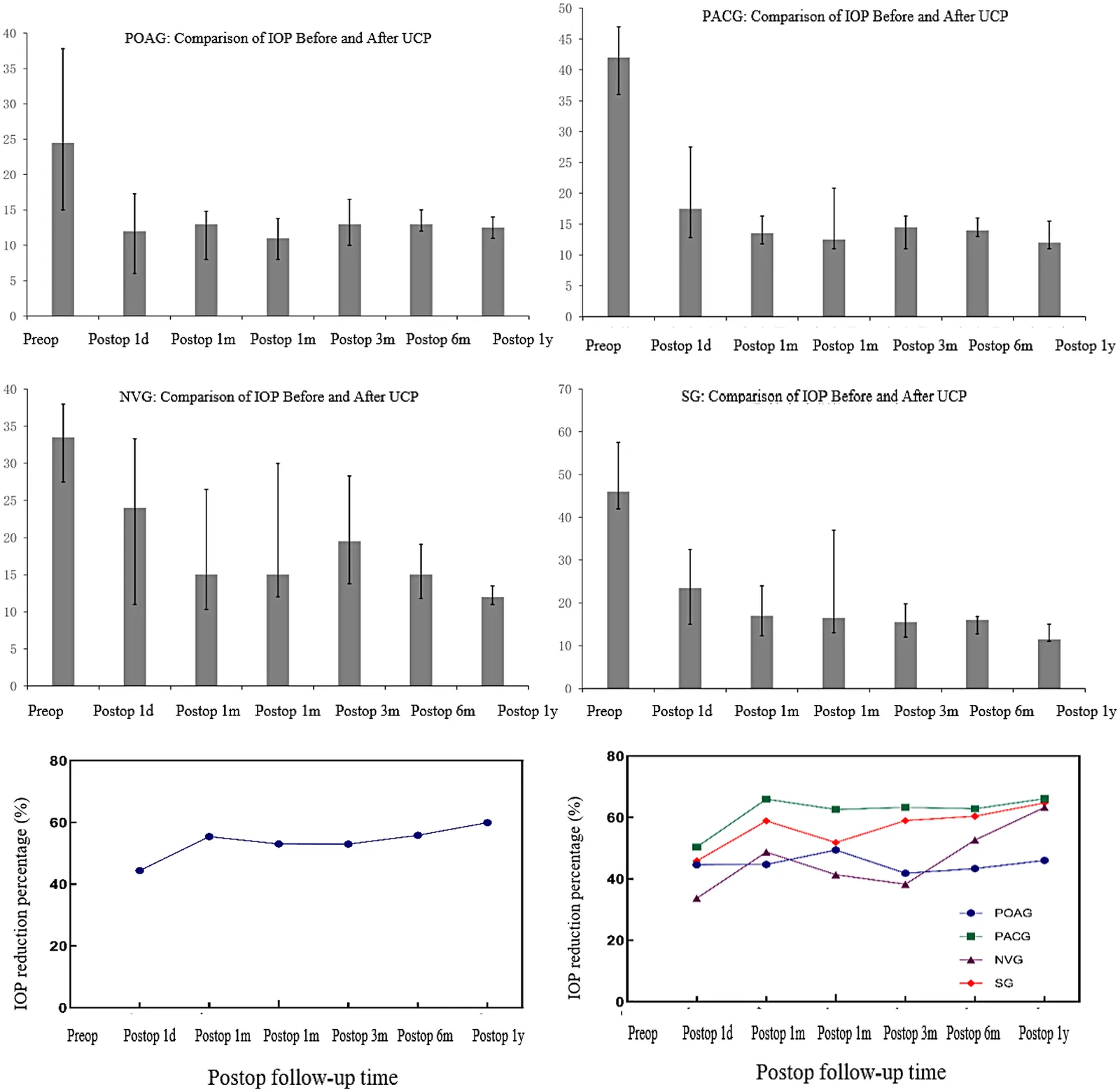
Percentage reduction of IOP after UCP overall and by subtypes.

### 3.4 Success rate of UCP

In this study of 30 patients undergoing UCP, postoperative follow-up revealed: The complete success rate (defined as achieving target intraocular pressure without adjunctive medications or interventions) was 16.67%, 36.67%, 50.00%, 73.33%, 93.33%, and 100% at 1 day, 1 week, 1 month, 3 months, 6 months, and 1 year, respectively. The qualified success rate (allowing medications to maintain target IOP) was 73.33%, 93.33%, 93.33%, 90.00%, 96.67%, and 96.67% at the same intervals. POAG: Complete success rates at each follow-up interval were 25%, 62.5%, 62.5%, 87.5%, 100%, and 100%, while qualified success rates were 75%, 87.5%, 100%, 87.5%, 100%, and 100%. PACG: Complete success rates progressed as 30%, 50%, 90%, 100%, 100%, and 100%, with qualified success rates consistently at 80% initially, then stabilizing at 100%. NVG: Complete success rates improved sequentially (25%, 50%, 50%, 75%, 100%, and 100%), whereas qualified success remained 75% until month 3 before reaching 100%.

For SG patients, postoperative success rates at each follow-up timepoint were as follows: Complete success rates: 25%, 37.5%, 37.5%, 87.5%, 87.5%, 87.5%. Qualified success rates: 75%, 100%, 87.5%, 87.5%, 87.5%, 87.5% (Figure 8). The results indicate that UCP achieved the highest overall surgical success rates at 6 months and 1 year postoperatively, both reaching 96.67%. Notably: PACG patients attained 100% success as early as 1 week postoperatively, maintaining this excellent outcome consistently. Both NVG and SG patients showed significant improvement in success rates by 6 months, with NVG patients achieving 100% success at both 6 months and 1 year. These findings demonstrate distinct efficacy profiles of UCP across different glaucoma subtypes: PACG patients exhibited the most stable and outstanding therapeutic outcomes. Comparatively, NVG and SG patients may require more intensive postoperative monitoring during the early phase due to delayed treatment response. The cumulative success rates through the Postop 1d to Postop 1y follow-up duration are presented in Figure 9.

**Figure 8:**
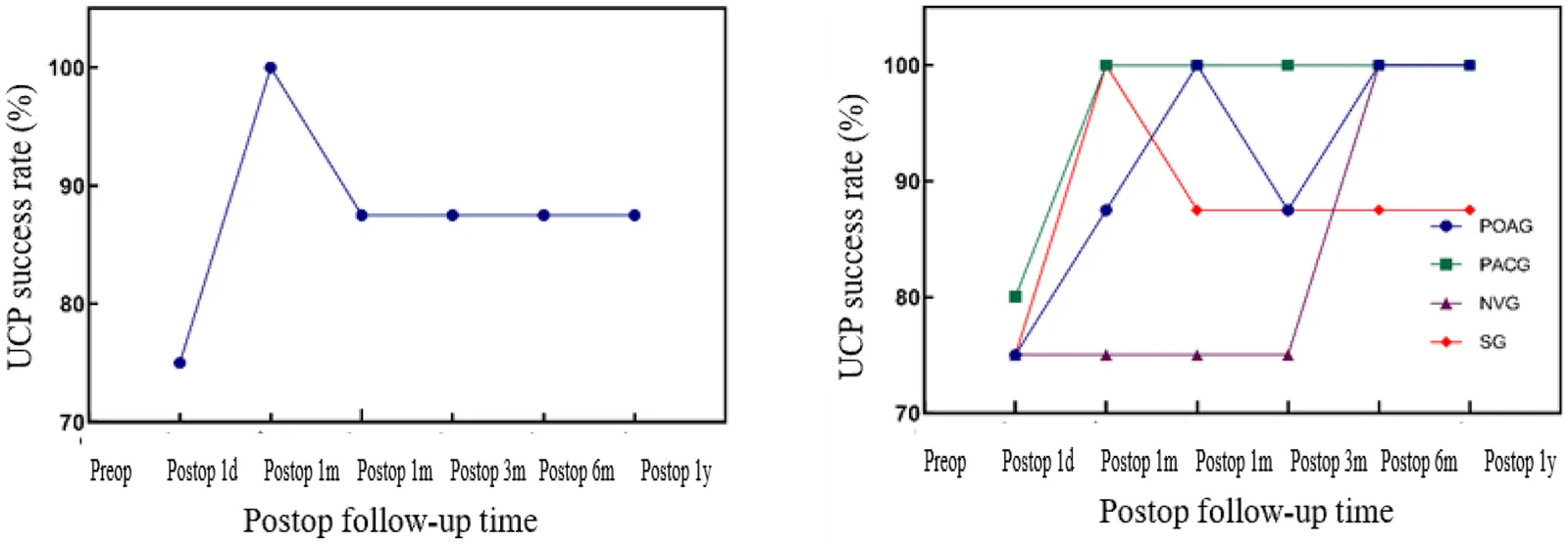
Surgical success rates (overall and by subtype) following UCP procedure.

**Figure 9:**
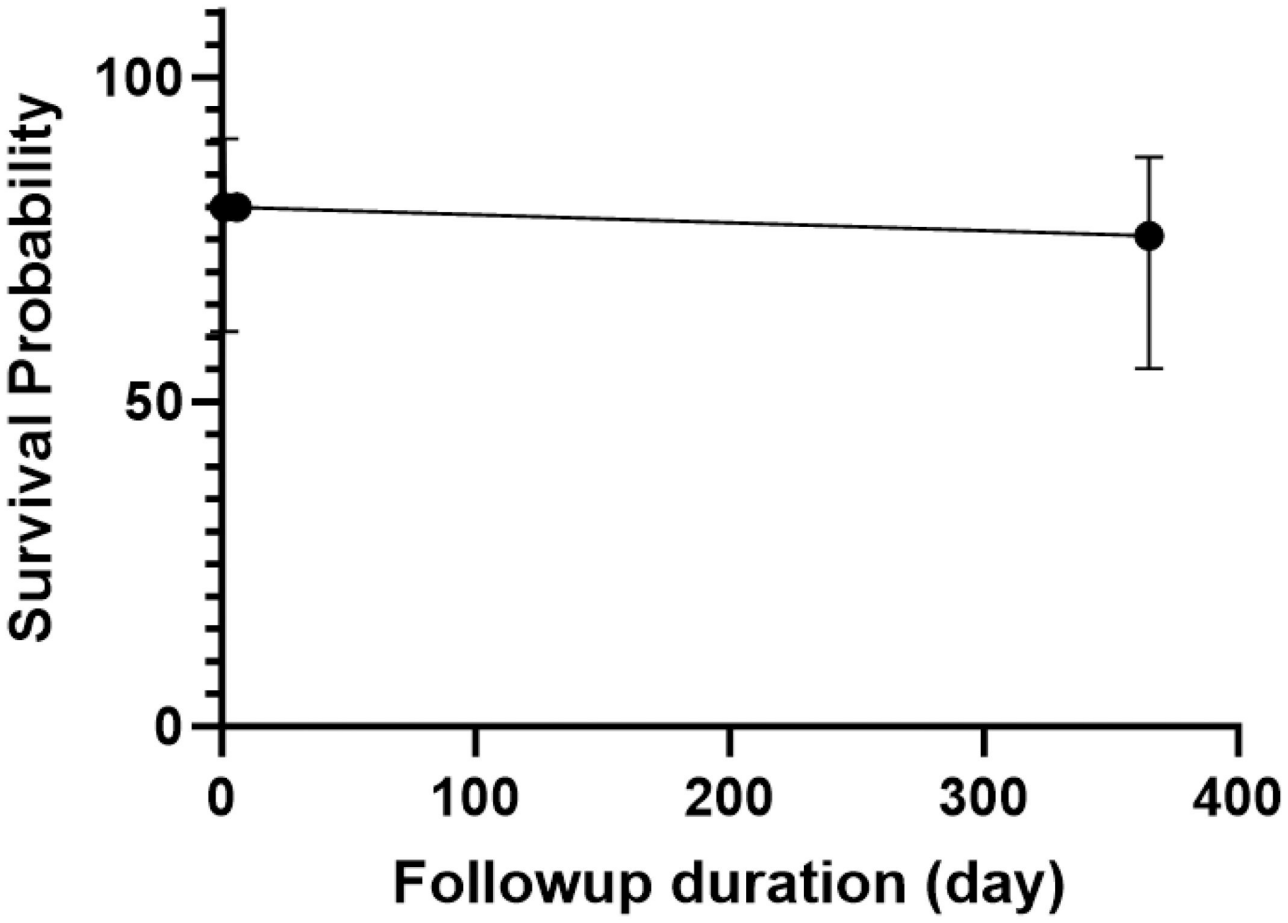
Kaplan–Meier analysis of efficacy maintenance time following UCP.

### 3.5 The correlation between the reduction in IOP at 1 year postoperatively and preoperative baseline IOP

The Spearman correlation coefficient between IOP reduction at 1 year postoperatively and preoperative baseline IOP was 0.641 (*P* < 0.01), indicating statistical significance. This demonstrates a positive correlation between preoperative IOP and the percentage of IOP reduction at 1 year postoperatively (see Figure 10 and Table 3).

**Figure 10:**
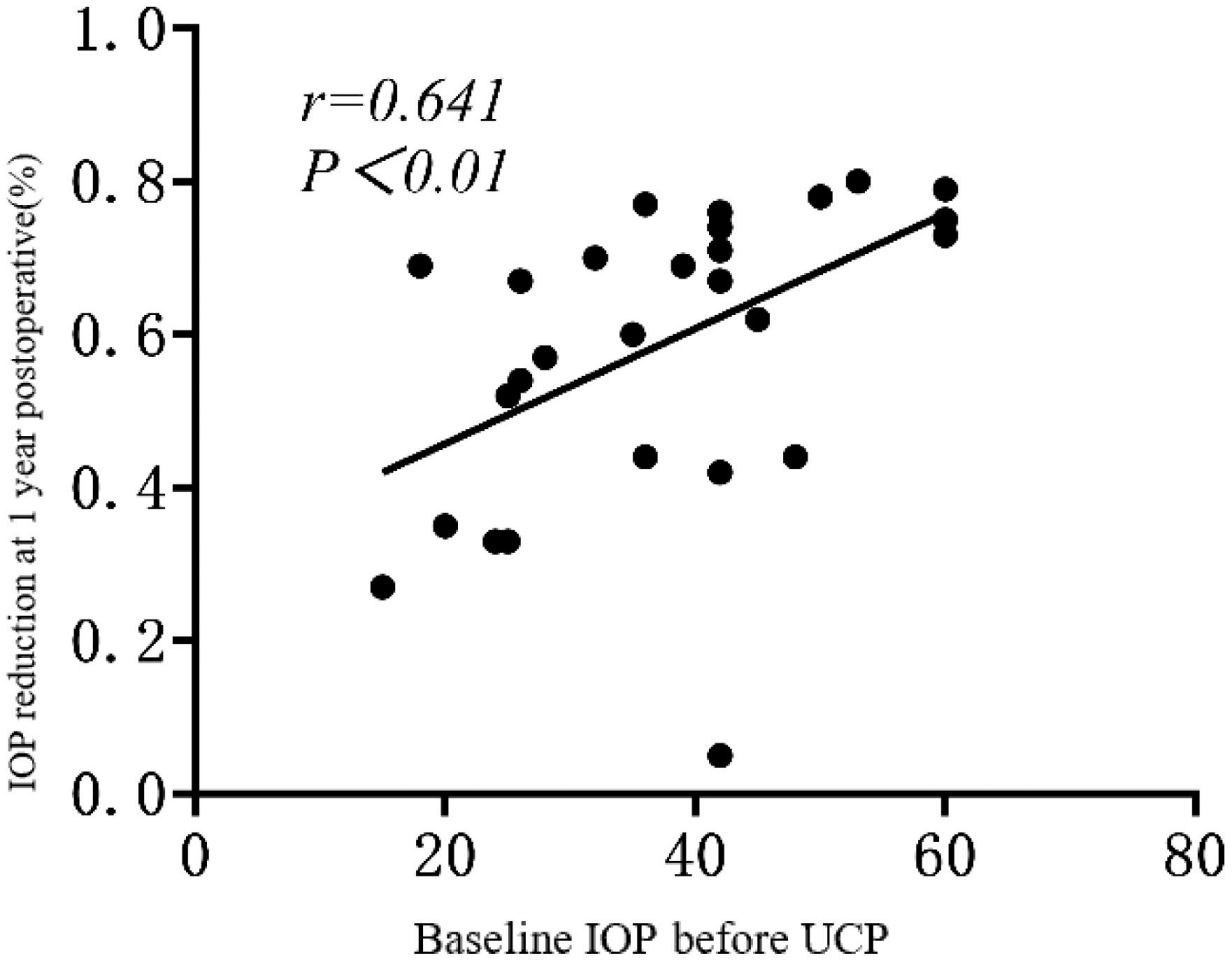
Relationship of baseline IOP with 1-year postoperative IOP reduction.

**Table 3:** Correlation between preoperative IOP and percentage of IOP reduction.

Variable	Preoperative IOP	Percentage of IOP Reduction
Preoperative IOP	1.000	0.641**
Percentage of IOP Reduction	0.641**	1.000

* Denotes *P* < 0.05.

** Denotes *P* < 0.01.

### 3.6 Comparison of MAPE before and after UCP

This study conducted nonparametric Wilcoxon signed-rank tests to analyze MAPE values in 19 eyes before and one day after UCP (ultrasound cycloplasty). Results demonstrated statistically significant differences (*P* < 0.05) between preoperative [10.6 ( 4.29, 14.5)] and postoperative day 1 MAPE values [5.3 ( 3, 7.75)]. The significant reduction in MAPE observed on postoperative day 1 indicates that UCP effectively stabilizes 24-hour intraocular pressure fluctuations in patients.

### 3.7 Pain score assessments

This study compared preoperative vs. 1-year postoperative pain scores using Wilcoxon signed-rank tests in 30 patients. Results showed median pain scores decreased significantly from 5 (3–7) preoperatively to 1 (1–2) at 1-year follow-up (*Z* = −4.558, *P* < 0.05). The negative *Z*-value confirms clinically meaningful pain reduction maintained at long-term follow-up, demonstrating sustained therapeutic efficacy of the intervention.

### 3.8 Complications

Postoperative complications following UCP were analyzed for incidence timing and resolution (Table 4), revealing:

**Table 4:** Incidence of postoperative complications following UCP.

Complication Type	N (%)	Timing of Occurrence
Intraoperative		
Eye pain	7 (23.3%)	Intraoperative
Postoperative		
Conjunctival hyperemia	26 (86.7%)	Within 24 h
Subconjunctival hemorrhage	8 (26.67%)	Within 24 h
Corneal edema	6 (20%)	Within 24 h
Anterior chamber inflammation	14 (46.7%)	Within 24 h
Pupil irregularity	12 (40%)	Within 48 h
Superficial punctate keratitis	4 (13.3%)	Within 24 h
Visual acuity decline	1 (3.33%)	After 2 months
Corneal ulcer	1 (3.33%)	After 3 months
Scleral indentation	3 (16.7%)	Within 24 h

#### 3.8.1 Intraoperative complications

Procedure: All surgeries were successfully completed. Intraoperatively, mild ocular pain was reported in 7 eyes (23.33%), manifesting as transient stinging or dull ache, which gradually subsided upon treatment completion.

#### 3.8.2 Postoperative inflammatory responses

##### Postoperative inflammatory response

Within 24 h postoperatively, 26 eyes (86.67%) developed conjunctival hyperemia, characterized by mild to moderate dilation of episcleral vessels, with or without subconjunctival hemorrhage (8 eyes, 26.67%; see Figure 11). The conjunctival congestion gradually subsided within 3 days, while the subconjunctival hemorrhage resolved completely within 7 days to 2 weeks. Additionally, 6 eyes (20%) exhibited mild corneal edema within 24 h, manifesting as epithelial edema and slight stromal haze, accompanied by mild visual blurring. These symptoms significantly improved by 1 week postoperatively and fully resolved within 2 weeks. At 48 h post-surgery, 14 eyes (46.67%) presented with anterior chamber inflammation, evidenced by increased aqueous flare and few keratic precipitates (KP)—without significant fibrinous exudation or posterior synechiae formation. Furthermore, 4 eyes (13.33%) developed superficial punctate keratitis within 24 h.

**Figure 11:**
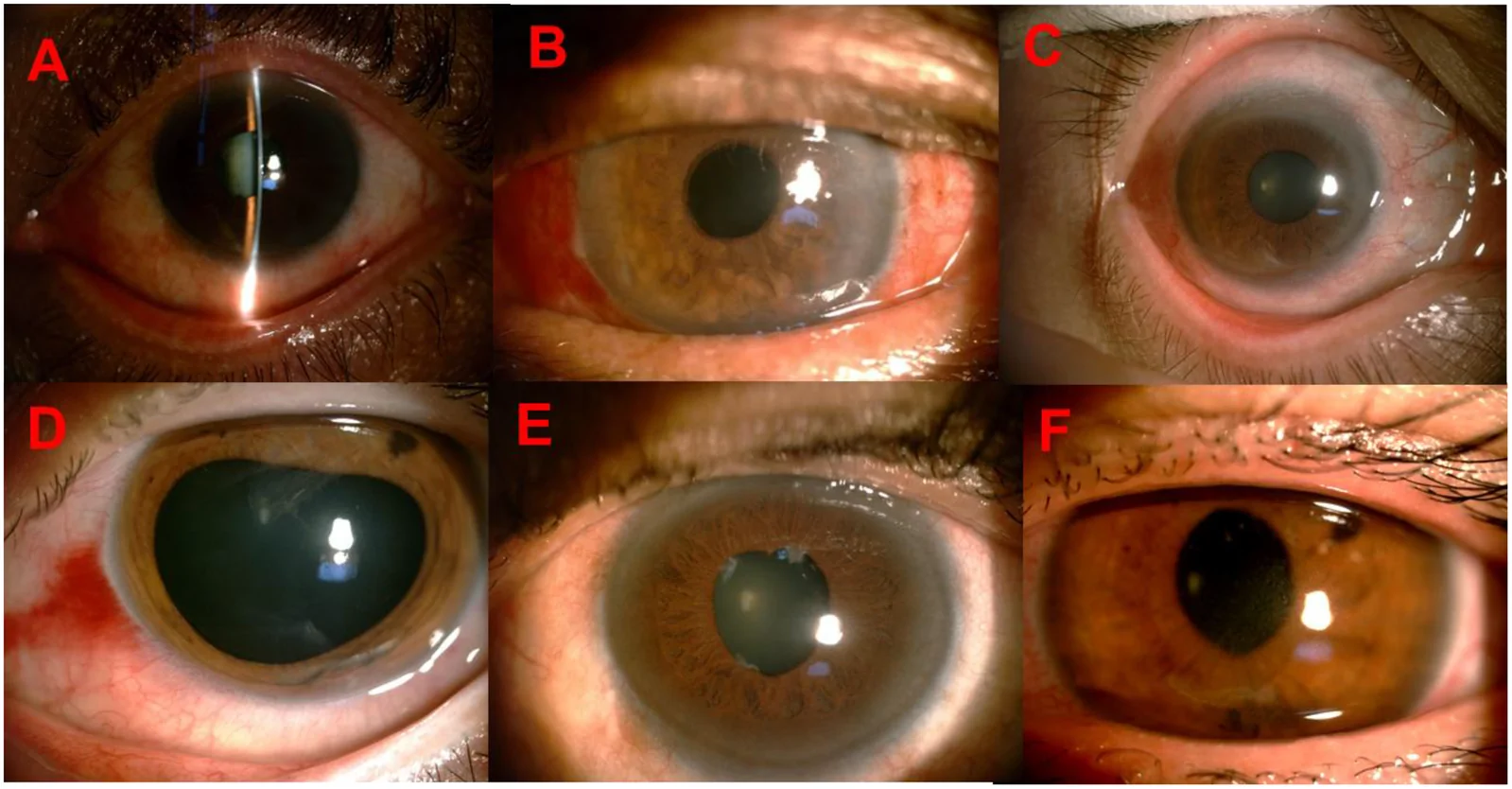
Typical complications before and after UCP surgery. **(A)** Conjunctival Hyperemia and Edema, **(B)** Corneal Edema, **(C)** Scleral Imprinting, **(D)** Anterior Chamber Inflammatory Reaction, **(E)** Pupil Irregularity, **(F)** Superficial Punctate Keratitis.

##### Postoperative pupillary changes

Post-UCP pupillary changes were observed in this cohort, with 40% of patients exhibiting postoperative mydriasis. Twelve eyes (40% of cases) demonstrated pupillary irregularities, manifesting primarily as mild pupillary margin unevenness accompanied by dilation; some cases additionally presented with slight corectopia while maintaining normal light reflexes. Following exclusion of cases showing severe anterior chamber inflammation (aqueous cells ≥3+), the remaining affected patients were treated with short-term miotic therapy using 0.5% pilocarpine eye drops. Subsequent follow-up at postoperative day 14 revealed stabilization of pupillary morphology in all treated eyes (3) Special Postoperative Complications:

At 2 months postoperatively, one patient (3.33%) experienced mild visual blurring. Extensive evaluation ruled out macular edema, secondary cataract, or other pathologies, with vision spontaneously recovering to baseline levels within 6 months. Additionally, one case (3.33%) developed a corneal ulcer at 3 months postoperatively, potentially attributable to corneal epithelial defects and postoperative dry eye. Following anti-inflammatory therapy and corneal repair treatment, the ulcer resolved completely within 1 month, leaving no corneal scarring.

#### 3.8.3 Long-term safety evaluation

Three eyes (16.67%) developed scleral imprints at the treatment site, manifesting as either localized mild pigmentation or focal scleral thinning, without significant intraocular pressure fluctuations or subjective discomfort. During follow-up, no cases exhibited serious complications such as profound hypotony (IOP ≤5 mmHg), choroidal detachment, macular edema, or phthisis bulbi. These observations indicate favorable long-term safety outcomes following UCP procedures.

## 4 Discussion

### 4.1 Epidemiological analysis of glaucoma in northwest China

Glaucoma, one of the leading global causes of blindness, affects approximately 64.3 million people worldwide (3.5% of the global population) according to epidemiological data (29)，Among these cases, about 5.7 million individuals suffer from visual impairment, while 3.1 million experience blindness due to the disease (30, 31). In China, the rapidly aging population has been accompanied by a steady annual increase in glaucoma prevalence. Notably, while Northwest China maintains a lower glaucoma prevalence compared to other regions, recent years have witnessed a progressive rise in both overall incidence and particularly striking increases in POAG rates (29). From 2000 to 2010, Northwest China experienced a 3.68% increase in POAG prevalence (29). Additionally, the number of PACG cases in Northwest China continues to exhibit a sustained upward trend. This underscores the growing importance of early screening, diagnosis, and treatment of glaucoma in the region. Therefore, research on the therapeutic efficacy of UCP in glaucoma patients in Northwest China holds significant clinical value. Such studies would not only optimize region-specific treatment strategies but also contribute to reducing visual impairment and, crucially, slowing disease progression in affected individuals.

### 4.2 Significant long-term IOP reduction efficacy and differential responses across glaucoma types

This study confirms that UCP provides significant long-term intraocular pressure (IOP)-lowering efficacy, with the maximum IOP reduction of 59.91% achieved at 12 months postoperatively. It is particularly noteworthy that a significant positive correlation was found between preoperative baseline IOP and postoperative IOP reduction. This finding aligns with the views of some researchers in the existing literature, who propose a positive correlation between baseline IOP levels and the magnitude of IOP reduction with UCP (9, 16, 26). Compared to previous studies, this study observed a more significant IOP reduction rate at the final follow-up, a finding that differs from the results reported by Giannaccare et al. (8, 16). We believe this discrepancy primarily stems from the generally higher baseline IOP levels of the patients included in this study. Mechanistically, pathological changes such as congestion and edema of the ciliary processes under conditions of high IOP may increase the sensitivity of ciliary body epithelial cells to ultrasonic energy, thereby achieving more pronounced IOP reduction through enhanced compensatory inhibition of aqueous humor production (32, 33). However, it is important to note that, despite the statistical significance of this correlation, we should remain cautious of the potential existence of a “ceiling effect”: when baseline IOP exceeds a certain threshold, the compensatory imbalance between aqueous production and outflow systems might diminish the effectiveness of UCP treatment (32).

Further analysis revealed that IOP changes postoperatively followed a consistent trend across different types of glaucoma. In POAG patients, IOP at 1 day, 1 week, 1 month, and 1 year postoperatively was significantly lower than preoperative levels, with the 1-month postoperative IOP showing the most pronounced decrease. This suggests that the 1-month postoperative period might be the time of most significant IOP decline in POAG, potentially related to the strongest inflammatory response and inhibition of aqueous production in the ciliary body during the early postoperative period. However, IOP at 3 and 6 months postoperatively showed no significant difference compared to preoperative IOP. This indicates that in POAG patients, postoperative IOP reduction may be more rapid and significant in the short term, but stabilizes over longer follow-up periods. This is consistent with the findings of Figus et al. in a previous clinical study on UCP for POAG, which reported the largest IOP reduction at 1 to 3 months post-UCP, followed by stabilization (34).

For PACG, the IOP reduction was most significant at 1 week postoperatively, suggesting that UCP treatment provides particularly prominent IOP control in PACG patients during the short-term postoperative period, especially at 1 week. The IOP reduction reached 66.12% at 1 year postoperatively, indicating sustained and stable long-term IOP control up to 1 year and further validating the efficacy and sustainability of UCP surgery for IOP management in PACG patients.

The reason for analyzing NVG separately from the broader category of SG in this study is its unique pathophysiological mechanism (retinal ischemia); its clinical characteristics and diagnostic urgency (early diagnosis, short treatment window, and frequent association with severe vision loss or blindness risk); and the role of UCP in NVG treatment, where it serves primarily as part of IOP reduction to create a time window for subsequent panretinal photocoagulation and anti-VEGF therapy (35). This study confirms that UCP effectively reduces IOP in NVG patients, with the most significant IOP-lowering effect observed at 1 year postoperatively (63.25% reduction). Analysis showed no statistically significant difference in IOP at 1 day, 1 month, and 3 months postoperatively compared to preoperative levels, which may be related to the early postoperative disruption of the blood-aqueous barrier, inflammatory response, and temporary obstruction of aqueous outflow (36). However, the overall IOP trend was downward. The results indicate that UCP provides short-term therapeutic effects in NVG and achieves sustained, stable IOP reduction in long-term follow-up. Since the number of cases in the NVG subgroup of this study was limited, we also observed a beneficial trend of intraocular pressure reduction in these patients based on preliminary findings. However, due to the extremely small sample size, these observations only serve as preliminary clues for future in-depth research in these specific glaucoma subtypes. The exact efficacy and therapeutic significance must be confirmed through well-designed subsequent studies with adequate sample sizes.

For SG, IOP showed a decreasing trend at 1 day, 1 week, 1 month, 3 months, 6 months, and 1 year postoperatively, gradually stabilizing. The IOP reduction was most significant at 1 year postoperatively, indicating a clear long-term effect of UCP in glaucoma patients.

The order of IOP reduction magnitude across glaucoma types was: PACG > SG > NVG > POAG. This ranking aligns with the conclusions of clinical studies by Giannaccare and Hu et al. (16, 26). The possible mechanisms for the most pronounced efficacy of UCP in PACG patients include: firstly, UCP effectively reduces angle pressure by decreasing aqueous humor production; secondly, its biomechanical effect of inducing scleral thinning may help open aqueous drainage channels (12); furthermore, the generally higher baseline IOP levels in angle-closure glaucoma patients also contribute to the more significant IOP-lowering effect observed with UCP (36).

### 4.3 High and stable surgical success rate and exploration of its contributing factors

This study demonstrates an overall success rate of 96.67% for UCP (Ultrasound Cyclo Plasty) at one-year postoperatively (final follow-up). This result significantly surpasses the rates reported in multiple key previous studies [e.g., Zhang et al.: 90.63% (43); Wang et al.: 81% (37); Almobarak et al.: 88.9% (38); Torky et al.: 77.4% (11); Wang et al.: 77.8% (39); Giannaccare et al.: 70% (17); Hu et al.: 52.9% (26)].

We believe the high success rate observed in this study may be closely attributed to the following factors. First: a rigorous preoperative examination and patient screening protocol. The use of comprehensive evaluations, including panoramic UBM and IOL-MASTER measurements, effectively selected glaucoma patients with high potential for a favorable UCP treatment response. Second: the implementation of a standardized surgical procedure. Specialized preoperative training for the surgeons ensured technical consistency throughout the intervention. Third: the establishment of strict, comprehensive perioperative management. This included 24-hour dynamic postoperative IOP monitoring, quantitative assessment of anterior chamber inflammation, and personalized adjustments to the IOP-lowering regimen, which effectively mitigated the occurrence of complications.

### 4.4 Innovative dynamic efficacy indicators

This study innovatively employed MAPE (mean absolute percentage error) as a dynamic monitoring indicator for evaluating the efficacy of UCP. At 1 day postoperatively, the MAPE was significantly lower than the preoperative value (*P* = 0.001), decreasing from 10.6 (4.29, 14.5) to 5.3 (3, 7.75). These results confirmed that UCP effectively reduces intraocular pressure (IOP) fluctuations and improves pressure stability, even in cases with isolated nocturnal IOP elevation. The MAPE indicator, screened via an SD (standard deviation) threshold, filters out minor physiological variations and specifically reflects pathological IOP drift. The rapid postoperative decline in MAPE suggests that UCP stabilizes IOP through a dual mechanism: (1) focused ultrasound thermo-coagulation suppresses aqueous humor production, and (2) collagen remodeling enhances the patency of outflow channels (30).

Typical case: A 49-year-old male with POAG, primarily presenting with nocturnal IOP elevation. Preoperatively, the MAPE was 12.3 in the right eye and 8.3 in the left eye. One day after UCP, the MAPE dropped to 6.7 in the right eye and 2.6 in the left eye, with a stable 24-hour IOP curve (Figure 12). This further validates the efficacy and sustained stabilizing effect of UCP on nocturnal IOP control.

**Figure 12:**
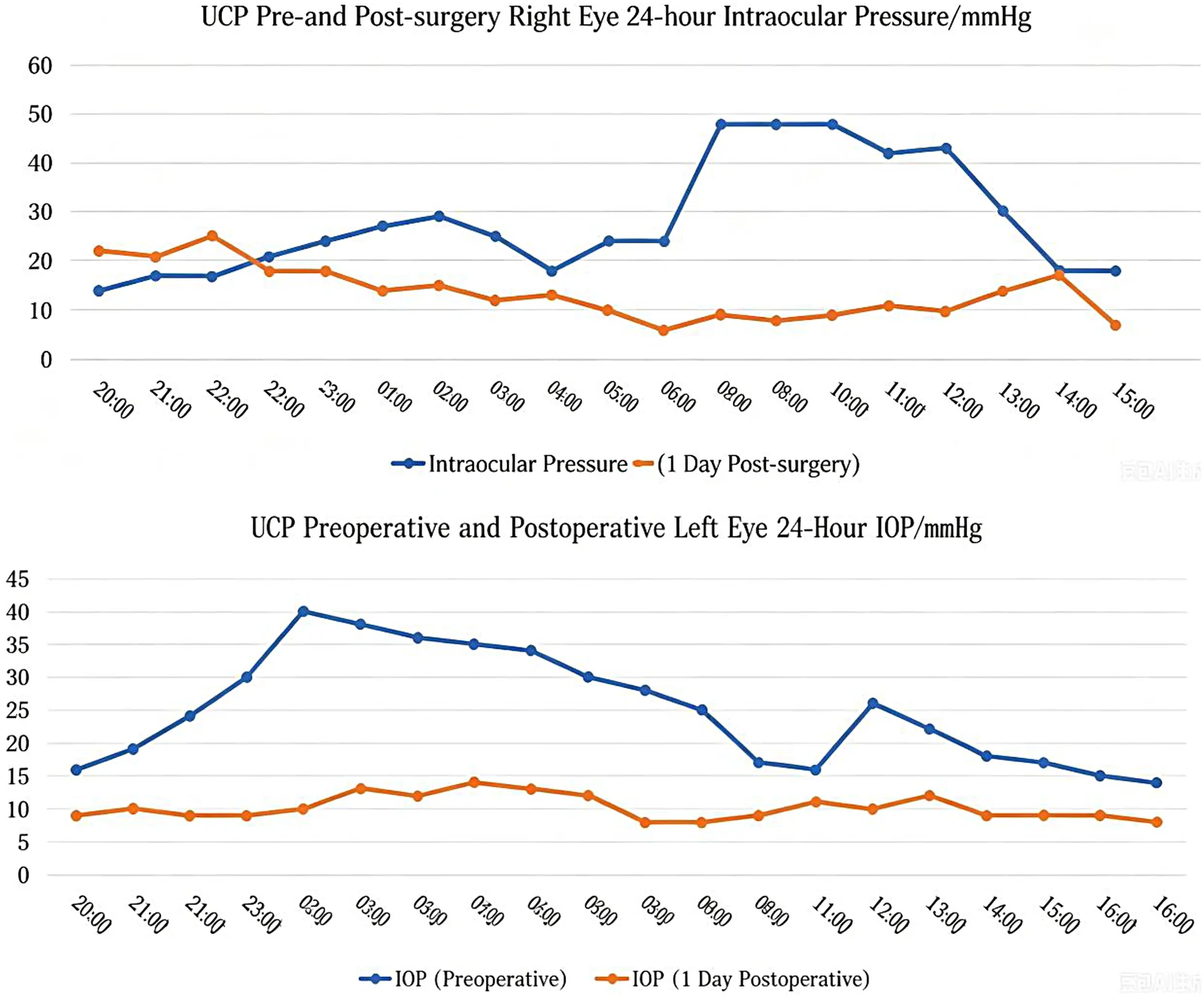
Comparison of 24-hour intraocular pressure curves before and after UCP surgery.

### 4.5 Acceptable safety profile and long-term improvement in patient-reported outcomes

This study analyzed the safety of UCP treatment, and the results indicate a generally favorable safety profile, as well as a long-term improvement in patient-reported pain symptoms. A comparison of pain scores between the 30 patients preoperatively and at 1-year postoperatively showed a statistically significant difference (*P* < 0.05), suggesting that UCP surgery may provide long-term relief from glaucoma-related pain. Pain alleviation is a key aspect of improved quality of life for patients. No severe complications such as choroidal detachment occurred during or after the procedure.

Common postoperative reactions primarily included: transient conjunctival hyperemia (86.7%), anterior chamber inflammation (46.7%), and changes in pupil morphology or reflex (40%). These reactions typically resolved spontaneously within several weeks or were stabilized with the application of non-steroidal anti-inflammatory drugs or corticosteroid combined with antibiotic eye drops.

Anterior chamber inflammation, presenting as increased aqueous flare and a few keratic precipitates without fibrin exudation or posterior synechia, was observed in 46.67% of patients within 24 h postoperatively. This may be attributed to cellular debris and protein fragments released from coagulative necrosis of ciliary epithelium during the procedure. Superficial punctate keratitis occurred in 13.33% of patients, requiring treatment with corneal nutritional support and anti-inflammatory medication, with symptoms resolving within 7 days to 2 weeks postoperatively. Corneal edema had an incidence of 20%, manifesting as corneal epithelial edema and mild stromal haze, but notably improved in all patients within 1 to 2 weeks postoperatively. Pupillary irregularity was noted in 40% of patients, primarily characterized by mild irregular pupillary margins, mydriasis (dilation), or slight displacement, along with a somewhat sluggish light reflex. Pupil morphology gradually stabilized during follow-up. Sousa et al. observed similar phenomena in their study, where patients exhibited fixed, dilated, and light-non-reactive pupils following UCP treatment, with these symptoms improving over time (40).

Other less common adverse reactions included diminished visual acuity and corneal ulceration. One patient experienced blurred vision within 2 months postoperatively. After ruling out macular edema and secondary cataract, this was speculated to be related to ultrasound energy affecting the pupillary sphincter muscle, causing mydriasis. The blurred vision resolved within 6 months postoperatively with the use of mydriatic (dilating) eye drops. Another patient developed a corneal ulcer 3 months postoperatively, possibly associated with corneal epithelial damage or dry eye syndrome. The ulcer healed within one month following anti-inflammatory and corneal repair treatment, without scar formation.

Compared to previous studies, the incidence of complications in this study was relatively lower. For instance, Aptel et al. reported a case of severe postoperative choroidal detachment which resolved completely after 2 months of treatment (14), whereas no such case occurred in this study. Furthermore, this study did not observe serious complications such as macular edema (41), cataract progression (42), or retinal detachment (26).

In summary, UCP demonstrates a favorable safety and efficacy profile in glaucoma treatment. Although postoperative reactions such as conjunctival hyperemia and corneal edema may occur, the associated complications are primarily mild to moderate and self-limiting, with a low incidence of severe complications. Simultaneously, this treatment shows potential for long-term relief of ocular pain in patients during postoperative follow-up. Collectively, these data indicate that UCP has an acceptable safety profile in glaucoma management.

## 5 Limitations and future directions

While this study demonstrated UCP's efficacy, several limitations should be addressed: 1. Sample size and follow-up duration, though sufficient for statistical analysis, require expansion—particularly for refractory glaucoma and NVG—to confirm long-term safety and efficacy. 2. Individual variability in treatment response correlated positively with baseline IOP; future multicenter studies should explore dose-effect relationships and predictive models using machine learning. 3. The data analysis may be subject to statistical bias due to the lack of between-eye correlation adjustment, which is relevant as many patients had both eyes enrolled. 4. Dynamic efficacy assessment was limited by short-term (24-hour) IOP monitoring due to patient compliance issues. We propose: (i) extending monitoring to 72 h in select patients, and (ii) simplifying protocols (e.g., daytime 5-point + home tonometry) to improve feasibility.

## Data Availability

The raw data supporting the conclusions of this article will be made available by the authors, without undue reservation.
